# Liposome Bupivacaine Compared to Plain Local Anesthetics to Reduce Postsurgical Pain: An Updated Meta-Analysis of Randomized Controlled Trials

**DOI:** 10.1155/2018/5710169

**Published:** 2018-07-15

**Authors:** Mark C. Kendall, Lucas Jorge Castro Alves, Gildasio De Oliveira

**Affiliations:** Department of Anesthesiology, Rhode Island Hospital, The Warren Alpert Medical School of Brown University, USA

## Abstract

**Objective:**

Peripheral nerve blocks for postoperative analgesia have improved block success, but analgesia efficacy has been limited by the short duration of traditional local anesthetics. The results of randomized trials comparing liposome bupivacaine with conventional local anesthetic formulations (e.g., plain bupivacaine and ropivacaine) have generated conflicting results. This study was conducted to systematically review the effectiveness of analgesic efficacy of liposome bupivacaine infiltration at the surgical site versus plain local anesthetic bupivacaine or ropivacaine in patients undergoing surgery.

**Methods:**

PRISMA statement guidelines were followed. A search of electronic databases National Library of Medicine's PubMed database, Cochrane Database of Systematic Reviews, Embase, and Google Scholar from January 2012 to September 2017 was performed. Among the 1,612 records identified, 9 randomized controlled trials involving 779 patients were eligible for data extraction and meta-analysis.

**Results:**

Liposome bupivacaine did not reduce postsurgical pain at rest compared to plain local anesthetics at 24 and 48 hours after surgery. Moreover, liposome bupivacaine did not reduce postoperative opioid consumption at 24, 48, or 72 hours when compared to plain local anesthetics. Liposome bupivacaine did reduce postoperative nausea when compared to plain local anesthetics (*P* =<0.3). There was no significant difference in hospital length of stay between study groups, the use of plain bupivacaine or ropivacaine, or among orthopedic or nonorthopedic procedures. No manifestations of local anesthetic toxicity were reported.

**Conclusions:**

Our results suggest that liposome bupivacaine does not have an analgesic advantage when compared to plain local anesthetics at the surgical site for patients undergoing surgical procedures.

## 1. Introduction

The management of postsurgical pain remains to be a challenge in patients undergoing surgery and is a major cause of patient dissatisfaction [1, 2]. In the past decade, the effort to reduce the severity of postsurgical pain has become a focal point for perioperative physicians [3, 4]. Improving management of postsurgical pain has shown to hasten patient recovery by initiating physical therapy sooner which ultimately leads to a shorter hospital stay and improved patient reported outcomes [5–7].

In the past decade, the placement of local anesthetics at the surgical site has become increasingly popular in the management of postoperative pain following surgery. The infiltrate usually consists of diluted local anesthetics often with nonlocal anesthetic adjuvants such as epinephrine or ketorolac. The analgesic duration may be prolonged with the placement of a catheter into the surgical field; however, it is usually surgery specific due to the surrounding anatomy.

Liposome bupivacaine, a multivesicular formulation of 1.3% bupivacaine, has been developed in order to improve analgesic duration of local anesthetics [8]. Liposome bupivacaine is approved for local administration use and as of February 2018, for the use in interscalene brachial plexus nerve blocks for shoulder surgery [9]. Liposome bupivacaine has not received approval for use in other peripheral nerve blocks for postsurgical analgesia. Nonetheless, the results of randomized trials comparing liposome bupivacaine with conventional local anesthetic formulations (e.g., plain bupivacaine, and ropivacaine) have generated conflicting results [10–12].

The main purpose of the current investigation is to investigate the analgesic efficacy of liposome bupivacaine infiltration at the surgical site versus plain local anesthetic bupivacaine or ropivacaine in patients undergoing surgery. We also sought to compare the side effects (safety profile) of liposome bupivacaine compared to plain local anesthetics in the same patient population.

## 2. Methods

We performed a quantitative systematic review following the guidelines of the Preferred Reporting Items for Systematic Reviews and Meta-Analyses statement [22]. All analyses were performed on previous published trials; therefore, institutional review board approval and patient consent were not required.

### 2.1. Systematic Search

Published publications of randomized trials evaluating the effects of bupivacaine extended-release liposome injection to local anesthetics bupivacaine or ropivacaine on postoperative surgical pain were searched using web-based literature involving the National Library of Medicine's PubMed database, the Cochrane Database of Systematic Reviews, Embase, and Google Scholar from January 2012 to September 2017.

Using free text, the terms ‘bupivacaine', ‘liposome bupivacaine', ‘Exparel', ‘extend-release', postsurgical, and ‘infiltration' were used in various combinations with no language restriction. The systematic investigation was limited to human participants greater than 18 years of age. An attempt to discover relevant studies that were not identified during the primary search was made by evaluating the reference lists from identified studies. No search was performed for unpublished studies. No minimum sample size was required for inclusion of the studies in the analysis. This initial screening yielded 1,612 randomized clinical trials.

### 2.2. Selection of Included Studies

Two authors (MCK and LJCA) independently reviewed the abstracts and results of the 1,612 articles obtained from the initial search using the predetermined inclusion and exclusion criteria. The trials that were not relevant were excluded. Any disagreements encountered during the selection process were resolved by discussion among the evaluators (MCK and LJCA). If there was a disagreement among the reviewers, then the final decision was resolved by the senior investigator (GDO).

### 2.3. Inclusion and Exclusion Criteria

We included randomized controlled trials that compared bupivacaine extended-release liposome injection at the surgical site with local anesthetic bupivacaine or ropivacaine in patients undergoing various surgical procedures. Studies that were identified to have an inactive (placebo or “no treatment”) control group were excluded. Studies containing a concurrent use of an alternative multimodal analgesia regimen were excluded if a direct comparison of liposome bupivacaine and plain bupivacaine could not be established. Nonrandomized controlled trials, letters, comments, or editorials were also not considered for inclusion. Included trials reported either on pain scores or opioid consumption as postoperative pain outcomes. No minimum sample size was required.

### 2.4. Data Extraction

Two authors (MCK, LJCA) independently evaluated the full manuscripts of all eligible studies. Data extraction was carried out by using a predesigned data collection form. Discrepancies between the two investigators (MCK and LJCA) were resolved by discussion. A third investigator (GDO) would serve as the final decision if the discussion among the investigators could not be reached. The variables obtained from trials included the sample size, local anesthetic type and dose, type of surgery, number of participants in treatment groups, 24, 48, and 72 hours postoperative pain scores, 24, 48, and 72 hours postoperative opioid consumption, postoperative nausea and vomiting, and length of hospital stay (hours). Postoperative opioid consumption was converted to the mg equivalents dose of oral morphine assuming no cross-tolerance (morEq) [23, 24]. Visual analog scale or numeric rating scale of pain was converted to a 0-10 Numeric Rating Scale (0 = no pain, 10 = extreme pain).

The data was initially extracted from either the text or tables. If the data could not be found in either location, then data was extracted manually from available figures. Continuous data was recorded using mean and standard deviation. Data presented only as median, interquartile range, or mean ± 95% confidence interval (CI) was converted to mean and standard deviation using previously described methodology [25]. When required, the standard deviation for pain scores was estimated using the most extreme values. If the same outcome was reported more than one time, the most conservative value was used.

### 2.5. Bias Assessment

The included studies were assessed in accordance with Cochrane Collaboration's tool for risk of bias assessment that includes the following six domains: selection bias, performance bias, attrition bias, detection bias, reporting bias, and other potential source of bias [26]. Each domain was recorded as “low risk”, “high risk”, or “Unclear risk” which indicates lack of information or unknown risk of bias. Two authors (MCK and LJCA) assessed the risk of bias of included studies independently. A third author was included in the assessment if there was a disagreement among the first two assessors (GDO).

### 2.6. Outcome Data

#### 2.6.1. Primary Outcomes

Postoperative pain scores (visual analog scale or numeric rating scale) at rest and opioid consumption (morEq) were reported at 24 hours following surgery.

#### 2.6.2. Secondary Outcomes

Postoperative pain scores (visual analog scale or numeric rating scale) at rest and opioid consumption (morEq) were reported at 48 and 72 hours following surgery. The length of hospital stay is presented in hours and postoperative nausea and vomiting as counts (n).

### 2.7. Meta-Analyses

The weighted mean differences (WMD) with 95% confidence interval (CI) were computed and reported for continuous data (Numeric Pain Rating Score (NRS) or Visual Analog Score (VAS) at rest at 24 h, total opioid consumption at 24 h, and length of hospital stay). A significant effect compared to control required that the 95% CI for continuous data did not include zero and for dichotomous data, the 95% confidence interval did not include 1.0. Due to the different surgical procedures, we choose to use the random effect model in an attempt to generalize our findings to studies not included in our meta-analysis [27].

In cases of significant effects, publication bias was investigated by examining for asymmetric funnel plots using Egger's regression test [28, 29]. A one-sided* P *< 0.05 was considered as an indication of an asymmetric funnel plot. In the case of an asymmetric funnel plot, a file drawer analysis was performed which estimates the lowest number of additional studies that if they would become available, it would reduce the combined effect to nonsignificance assuming the average z-value of the combined* P* values of these missing studies would be 0 [30].

Of the included studies, the heterogeneity was further analyzed if the I^2^ statistic was greater than 50%. Additional analysis was planned a priori to explore nontrivial heterogeneity of the treatment effect across the included studies. Subgroup analysis was performed to test if the overall effect of the liposome bupivacaine on evaluated outcomes changed when the drug in comparison was plain bupivacaine or ropivacaine. The types of surgery subgroups were also investigated to compare the effect of liposome bupivacaine in orthopedic to nonorthopedic procedures.

The proportion of the total variance explained by the covariates (R2) was calculated by dividing the random-effects pooled estimates of variance (Tau squared) within studies by the total variance (total Tau squared). The value obtained was then subtracted from 1. When values fall outside the range of 0 to 100%, they were set to the closest value (0% or 100 %). A P value < 0.05 was required to reject the null hypothesis and to minimize the chance of Type I error.

Analysis was performed using Stata version 13 (College Station, Texas) and Comprehensive Meta-analysis software version 3 (Biostat, Englewood, NJ).

## 3. Results

**Figure 1:**
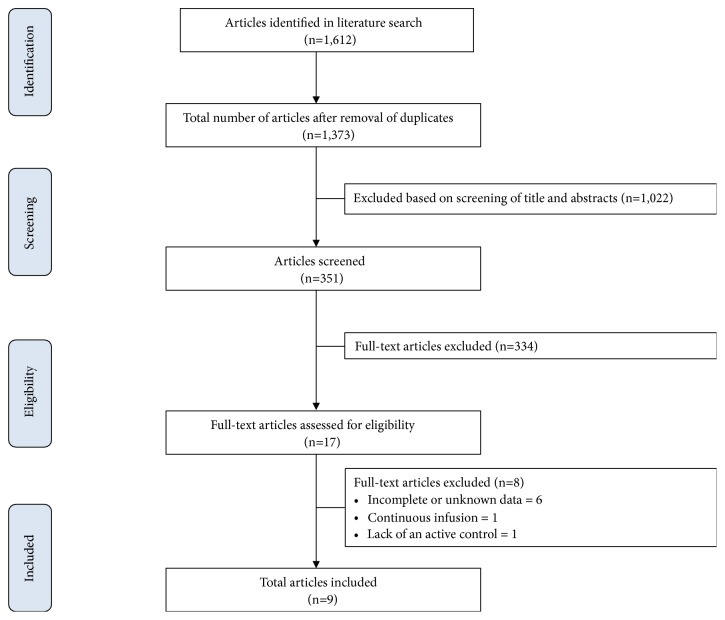
Flowchart of the selection of studies.

**Figure 2:**
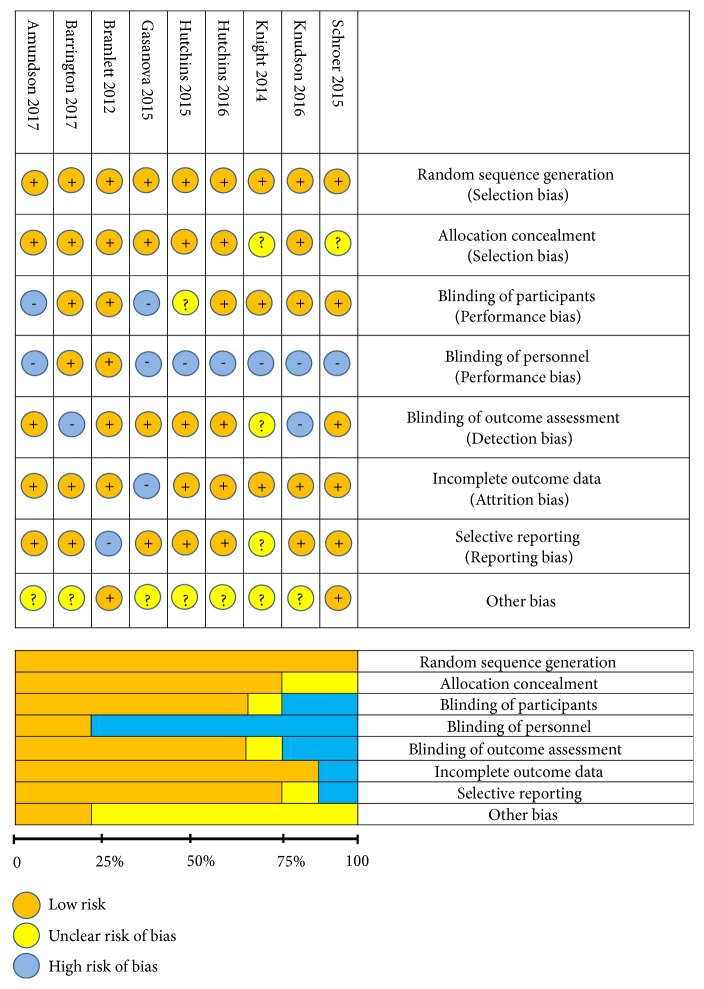
Risk of bias summary and bias graph.

Seventeen studies met the inclusion criteria (Figure 1). Eight studies were excluded due to not reporting complete data on evaluated outcomes, use of a continuous infusion control, or lack of an active control (e.g., bupivacaine or ropivacaine) [13, 31–37]. The risk of bias of included trials is presented in Figure 2. The characteristics of included trials are listed in Table 1. The evaluated trials included data from 779 subjects and were published between 2012 and 2017 [14–21, 38]. The median and interquartile range (IQR) number of patients in the included studies receiving liposome bupivacaine was 33 (29 to 54). All 9 studies reported on pain scores and/or opioid consumption.

## 4. Primary Outcome

### 4.1. Pain at Rest 24 h following Surgery

**Figure 3:**
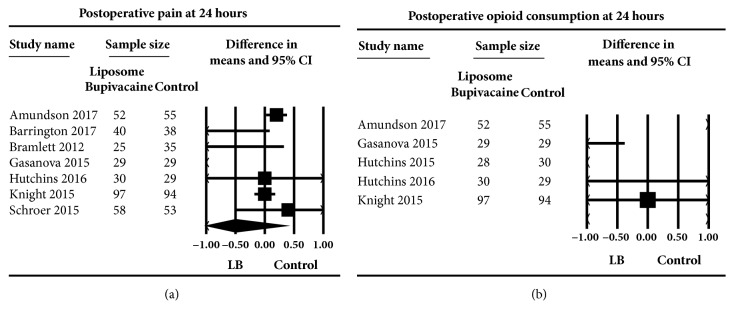
Primary outcome. Meta-analysis evaluating the effect of liposome bupivacaine on postoperative pain (a) and opioid consumption (b) compared to control at 24 hours following surgery. The overall effect of liposome bupivacaine versus control was estimated as a random effect. In part (a), the point estimate (95% confidence interval) for the overall effect was -0.50 (-1.37 to 0.37) (*P*=0.26) (0-10 numerical scale). In part (b), the point estimate (95% confidence interval) for the overall effect was -5.84 -16.86 to 5.18) (*P*=0.30) mg oral morphine equivalents. The weighted mean difference for individual studies is represented by the square symbol on Forrest plot, with 95% CI of the difference shown as a solid line. The size of the square and the thickness of the 95% CI line resemble the sample size.

The overall effect of 7 studies [14–17, 19, 20, 38] that examined the effect of liposome bupivacaine on postsurgical pain at rest compared to plain local anesthetics did not reveal a significant effect in relation to a large confidence interval, WMD (95% CI) of -0.50 (-1.37 to 0.37) (0-10 numerical scale), (*P* = 0.26) (Figure 3(a)). Heterogeneity was high (I^2^=98.12) and could be partially explained by orthopedic procedures (I^2^ = 62.40). The subgroup analysis revealed no effect on pain with liposome bupivacaine compared to plain bupivacaine, WMD (95%CI) of -0.55 (-1.75 to 0.66) versus liposome bupivacaine compared to ropivacaine WMD (95%CI) of -0.31 (-1.56 to 0.93),* P *= 0.62. When the type of surgery was evaluated, there was no difference on pain effect between orthopedic procedures WMD (95%CI) of -0.19 (-0.87 to 0.49) compared to nonorthopedic procedures WMD (95%CI) of -0.67 (-2.22 to 0.87),* P *= 0.40.

### 4.2. Postoperative Opioid Consumption 24 Hours following Surgery

The aggregated effect of five studies [14, 17–20] evaluating the effect of liposome bupivacaine on postoperative opioid consumption compared to control at 24 hours following surgery did not reveal a significant effect relative to a large confidence interval, weighted mean difference WMD (95% CI) of -5.84 (-16.86 to 5.18) morEq (*P *= 0.30) (Figure 3(b)). Heterogeneity was high (I^2^ = 78.12). Heterogeneity could be partially explained by the use of bupivacaine and ropivacaine as control groups (heterogeneity decreased (I^2^ = 46.19 for studies using bupivacaine alone)).

A subgroup analysis revealed no effect on opioid consumption with plain bupivacaine used a s control,WMD (95%CI) of -9.70 (-19.54 to 0.14) morEq when compared to ropivacaine used as control, WMD (95%CI) of 7.00 (1.88 to 12.12),* P *= 0.14. When investigating the type of surgery, there was no difference between opioid consumption for orthopedic procedures, WMD (95%CI) of 7.00 (1.88 to 12.12) morEq compared to nonorthopedic procedures WMD (95%CI) of -9.70 (-19.54 to 0.14) morEq,* P *= 0.14.

## 5. Secondary Outcomes

### 5.1. Pain at Rest 48 h following Surgery

**Figure 4:**
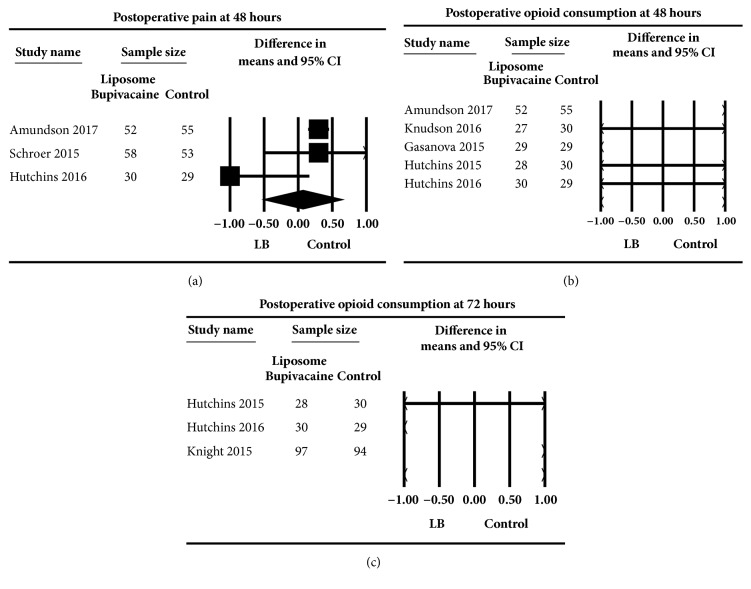
Secondary outcomes. The meta-analysis evaluating the effect of liposome bupivacaine on pain scores (a) and opioid consumption (b) at 48 hours and 72 hours (c) compared to control was estimated as a random effect. The point estimate (95% confidence interval [CI]) for the overall effect on postoperative pain scores at 48 hours following surgery was 0.07 (-0.51 to 0.66) (*P*=0.81), (0-10 numerical scale). The point estimate (95% CI) for the overall effect on postoperative opioid consumption at 48 hours following surgery was –3.57 (-14.83 to 7.70) (*P*=0.54) mg oral morphine equivalents. At 72 hours following surgery, the point estimate for opioid consumption was 2.44 (-43.08 to 47.96) (*P*=0.92) mg oral morphine equivalents. The weighted mean difference for individual studies is represented by the square symbol on Forrest plot, with 95% CI of the difference shown as a solid line. The size of the square and the thickness of the 95% CI line resemble the sample size. The diamond represents the pooled estimate and uncertainty for the effects of liposome bupivacaine compared to control.

The effect of the three studies [14, 19, 38] evaluating the effect of liposome bupivacaine on postsurgical pain compared to control at 48 hours following surgery did not demonstrate a significant effect, WMD (95% CI) of 0.07 (-0.51 to 0.66) (0-10 numerical scale),* P* = 0.81. (Figure 4(a)). Heterogeneity was moderate (I^2^=58.07). Subgroup analysis demonstrated a larger effect on pain when plain bupivacaine was used as control, WMD (95% CI) of -0.28 (-1.54 to 0.99) compared to when ropivacaine was used as control, WMD (95% CI) of 0.30 (0.15 to 0.45),* P* = <0.01. Furthermore, an analysis to examine variations on the effect by type of surgery revealed a significant difference between orthopedic procedures WMD (95% CI) of 0.30 (0.16 to 0.45) (0-10 numerical scale) compared to nonorthopedic procedures WMD (95% CI) of -1.0 (-2.16 to 0.16), (0-10 numerical scale),* P *= <0.01. Nonetheless, both differences (e.g., local anesthetic and type of surgery) were not clinically significant.

### 5.2. Postoperative Opioid Consumption 48 Hours following Surgery

The effect of five studies [14, 17–19, 21] evaluating liposome bupivacaine infiltration on postoperative opioid consumption compared to control did not reveal an effect WMD (95% CI) of -3.57 (-14.83 to 7.70) morEq,* P* = 0.54 (Figure 4(b)). Heterogeneity was high (I^2^=86.90). Subgroup analysis revealed a larger effect on opioid consumption when plain bupivacaine was used as control, WMD (95% CI) of -8.42 (-13.36 to -3.47) morEq, compared to when ropivacaine was used as control, WMD (95% CI) of 8.00 (4.75 to 11.26), morEq,* P* = < 0.03. Furthermore, a subgroup analysis to examine effect variations by type of surgery revealed a difference between orthopedic procedures WMD (95% CI) of 8.0 (4.75 to 11.26) morEq compared to nonorthopedic procedures WMD (95% CI) of -8.42 (-13.36 to -3.47), morEq,* P* = < 0.03.

### 5.3. Postoperative Opioid Consumption 72 Hours following Surgery

Three studies [18–20] evaluated the effect of liposome bupivacaine on postoperative opioid consumption compared to control and did not demonstrate an effect on opioid consumption at 72 hours compared to control, WMD (95% CI) of 2.44 (-43.08 to 47.96) morEq,* P *= 0.92 (Figure 4(c)). Heterogeneity was high (I^2^=99.28). All three studies examined nonorthopedic procedures and plain bupivacaine was utilized as control.

### 5.4. Length of Hospital Stay following Surgery

The aggregated effect of six studies [14, 15, 19–21, 38] evaluating the effect of liposome bupivacaine on length of hospital stay (hours) compared to control did not show an effect on length of stay relative to a large confidence interval, WMD (95% CI) of -1.44 (-3.81 to 0.93, hours,* P *= 0.23. Heterogeneity was moderate, I^2^=49.94. The heterogeneity decreased to I^2^ = 27.68% for orthopedic procedures. Subgroup analysis revealed no effect on hospital length of stay when plain bupivacaine was used as control, WMD (95%CI) of -3.27 (-7.91 to 1.38) hours compared to when ropivacaine was used as control, WMD (95%CI) of 0 (-2.21 to 2.21) hours,* P* = 0.56. In addition, a subgroup analysis to examine variation on the effect size by type of surgery did not detect a significant difference between orthopedic procedures, WMD (95%CI) of - 0.55 (-2.49 to 1.38) compared to nonorthopedic procedures, WMD (95%CI) of -4.97 (-14.88 to 4.95),* P* = 0.46.

### 5.5. Safety Analysis

Local anesthetic toxicity: no clinical symptoms of local anesthetic toxicity were reported among the included studies.

**Figure 5:**
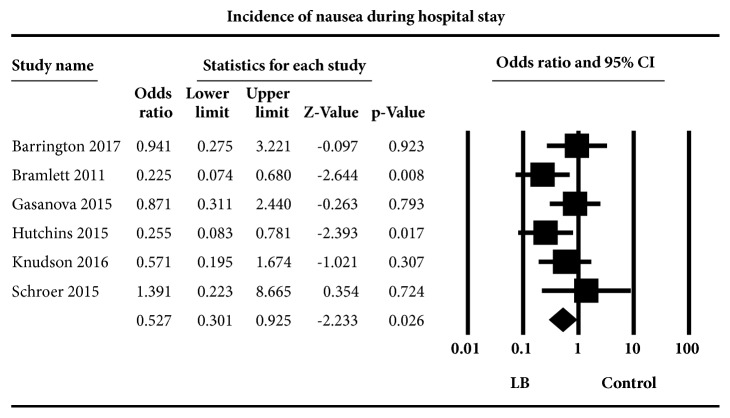
Incidence of nausea during hospital stay. Random-effects meta-analysis evaluating the effect of liposome bupivacaine on nausea compared to control. Squares to the right of the middle vertical line indicates that local anesthetics was associated with increased odds of nausea, whereas squares to the left of the middle vertical line show that liposome bupivacaine was associated with decreased odds of nausea. The horizontal lines represent the 95% CI and the diamond shape represents the overall effect of liposome bupivacaine on postoperative nausea compared to standard local anesthetics. CI = confidence interval.

Postoperative nausea and vomiting: in the six studies [15–18, 21, 38] that reported on nausea, the aggregated effect of the studies that investigated liposome bupivacaine on postoperative nausea compared to standard local anesthetics revealed a significant effect OR (95%CI) of 0.53 (0.30 to 0.93) (*P*=0.03) (Figure 5). Heterogeneity was low, I^2^=26.04. Subgroup analysis revealed an impact on nausea when plain bupivacaine was used as control, OR (95%CI) of 0.47 (0.25 to 0.89) compared to when ropivacaine was used as control, OR (95%CI) of 0.94 (0.28 to 3.22) counts,* P* = 0.03. A subgroup analysis to investigate the effect caused by type of surgery revealed a difference between orthopedic procedures OR (95%CI) of 0.59 (0.19 to 1.81) compared to nonorthopedic procedures OR (95%CI) of 0.52 (0.26 to 1.04),* P* = <0.04.

## 6. Conclusions

The most important finding of the current investigation is the lack of a clinically significant effect of liposome bupivacaine on postsurgical analgesia when compared to plain local anesthetics. Liposome bupivacaine did not reduce postoperative pain when compared to plain local anesthetics at 24 or 48 hours after surgery. In addition, liposome bupivacaine did not reduce postoperative opioid consumption at 24, 48, and 72 hours when compared to plain local anesthetics. Taken together, our results suggest that liposome bupivacaine does not have an analgesic advantage when compared to plain local anesthetics for patients undergoing surgical procedures.

Our results are clinically important since liposome bupivacaine can be mistakenly trusted by clinical practitioners to reduce late postoperative pain (≥24 hours). This may result in reduced use of more conventional multimodal analgesics with established efficacy in decreasing postoperative pain (e.g., nonsteroidal anti-inflammatory drugs, and acetaminophen) [39–42]. Moreover, the use of liposome bupivacaine may reduce the use of nerve or field blocks with established efficacy in improving postoperative analgesia [43–46].

Another relevant finding was the lack of a beneficial effect of liposome bupivacaine on hospital discharge times. Although this is an interesting discovery, discharge times are impacted by many variables such as postoperative pain, emetic symptoms, and system-related delays that was not included in our statistical model. Further exploration is warranted because liposome bupivacaine is considerably more expensive than plain bupivacaine or ropivacaine [47, 48] and may not have a pharmacoeconomic advantage when compared to plain bupivacaine or ropivacaine [49, 50].

It was interesting to note a significant effect of liposome bupivacaine on the reduction of postoperative nausea when compared to plain local anesthetics. Since we could not detect an effect of liposome bupivacaine on postoperative opioid consumption, the reduction of postoperative nausea cannot be attributed to opioid sparing effects [51, 52]. Since the reduction of postoperative nausea is an important goal for perioperative patients, the mechanism responsible for the antiemetic effect of liposome bupivacaine remains to be determined [53–55]. We also detected a reduction on heterogeneity when we evaluated a single type of surgical procedures (e.g., orthopedics). These findings support the concept of evaluating the benefits of analgesic interventions for a specific surgical specialty and, whenever possible, for a specific surgical procedure [56–58].

The findings of our systemic review should be interpreted within the context of several limitations. The high heterogeneity (>75%) that we observed in many of our analysis may be partially explained by the type of surgical procedure or by the type of plain local anesthetic used as control. Our subgroup analysis (orthopedic versus nonorthopedic procedures) should be interpreted as observational in nature and as hypothesis generating for future studies as only a large randomized trial can confirm or dispute our findings. None of the included studies evaluated the incidence of persistent postsurgical pain; therefore, we could not evaluate the effects of liposome bupivacaine on the incidence of persistent postsurgical pain.

In summary, we did not detect a beneficial effect of liposome bupivacaine on postsurgical analgesic outcomes when compared to plain local anesthetics. Clinical practitioners should not rely on liposome bupivacaine as a multimodal analgesic strategy to minimize postoperative pain rather than using established analgesic interventions.

## Figures and Tables

**Table 1:** Summary of study characteristics included in analysis.

**Authors**	**Year of Publication**	**Procedures**	**Number treatment**	**Treatment**	**Type of Anesthesia**	**Postoperative analgesia**	**Method of extraction†**
Amundson et. al [13]	2017	Total Knee Arthroplasty	52/55	Exparel 20 mL (266mg) +100mL NS 120 mL (300mg) Ropivacaine	Spinal or GA	Not standardized	Text Table

Barrington et. al [14]	2016	Total Knee Arthroplasty	40/38	Exparel 20 mL (266mg) + 10mL NS 50mL 0.5% Ropivacaine	Spinal	Unknown	Text Table

Bramlett et. al [15]	2012	Total Knee Arthroplasty	25/35	Exparel 20 mL (266mg) + 40mL NS .50% Bupivacaine 30mL + 30ml NS	General	1000mg Acetaminophen q tid IV-PCA morphine – no basal rate Oxycodone 5mg-10mg q 4-6h	Text Table

Gasanova et. al [16]	2015	Open Total Abdominal Hysterectomy	29/29	Exparel 20 mL (266mg) + 40mL NS Bilateral TAP Bupivacaine 0.5% 20mL	General	Hydromorphone 0.1 to 0.2mg IV until VAS <3 IV-PCA morphine 1mg bolus 5min lockout Ketrolac IV 30mg Acetaminophen 1 g PO q 6h After 24hrs – Acetaminophen 1 g PO q 8h Hydrocodone 5mg/Acetaminophen 325mg 1 to 2 tabs q6 as needed.	Text Figure

Hutchins et. al [17]	2015	Robotic assisted hysterectomy	28/30	Exparel 10 mL (266mg) + 20mL NS times 2 (bilateral) 0.25% Bupivacaine 30mL + epi 200K times 2 (bilateral)	General	Not detailed IV morphine eq only Ibuprofen Acetaminophen	Text Table

Hutchins et. al [18]	2016	Laparoscopic Hand-assisted nephrectomy	30/29	Exparel 10 mL (266mg) + 20mL NS 0.25% Bupivacaine 30mL (bilateral)	General	IV hydromorphone Fentanyl Hydromorphone PO Hydrocodone PO Oxycodone PO	Text Table

Knight et. al [19]	2015	Laparoscopic Urologic Surgery	97/94	Exparel 20 mL (266mg) + 40mL NS 0.25% Bupivacaine 60mL	General	Hydrocodone/Acetaminophen IV morphine	Text Figure

Knudson et. al [20]	2016	Colon Resection	27/30	Exparel 20 mL (266mg) + 10mL NS Bupivacaine 0.5% 30mL	General	Hydromorphone-Patient Controlled analgesia pump 5mg/325mg hydrocodone/acetaminophen as needed	Text Table Figure

Schroer et. al [21]	2015	Total Knee Arthroplasty	58/53	Exparel 20 mL (266mg) + 30mL 0.25% Bupivacaine 0.25% Bupivacaine 60mL	Spinal	Celecoxib 400mg daily Oxycontin 10mg q 12h IV-PCA morphine	Text Table
